# Inhibition of Human Respiratory Influenza A Virus and Human Betacoronavirus-1 by the Blend of Double-Standardized Extracts of *Aronia melanocarpa* (Michx.) Elliot and *Sambucus nigra* L.

**DOI:** 10.3390/ph15050619

**Published:** 2022-05-17

**Authors:** Michał Ochnik, Dominika Franz, Maciej Sobczyński, Piotr Naporowski, Mariusz Banach, Beata Orzechowska, Marta Sochocka

**Affiliations:** 1 Laboratory of Virology, Department of Immunology of Infectious Diseases, Hirszfeld Institute of Immunology and Experimental Therapy, Polish Academy of Sciences, 53-114 Wroclaw, Poland; michal.ochnik@hirszfeld.pl (M.O.); dominika.franz@hirszfeld.pl (D.F.); beata.orzechowska@hirszfeld.pl (B.O.); 2 Laboratory of Molecular Neurobiology, Nencki Institute of Experimental Biology of the Polish Academy of Sciences, 02-093 Warsaw, Poland; macsebsob@poczta.onet.pl; 3 Laboratory of Medical Microbiology, Department of Immunology of Infectious Diseases, Hirszfeld Institute of Immunology and Experimental Therapy, Polish Academy of Sciences, 53-114 Wroclaw, Poland; piotr.naporowski@hirszfeld.pl; 4 Department of Physical Chemistry and Polymer Physical Chemistry, Nicolaus Copernicus University in Toruń, 87-100 Toruń, Poland; m.banach@doktorant.umk.pl

**Keywords:** antiviral activity, inhibition of replication, extract of *Aronia melanocarpa*, extract of *Sambucus nigra*, human betacoronavirus, human influenza virus

## Abstract

Viral and bacterial diseases are among the greatest concerns of humankind since ancient times. Despite tremendous pharmacological progress, there is still a need to search for new drugs that could treat or support the healing processes. A rich source of bioactive compounds with antiviral potency include plants such as black chokeberry and elderberry. The aim of this study was to assess the in vitro antiviral ability of an originally designed double-standardized blend of extracts from *Aronia melanocarpa* (Michx.) Elliot and *Sambucus nigra* L. (EAM-ESN) or separated extracts of *A. melanocarpa* (EAM) or *S. nigra* (ESN) against four human respiratory tract viruses: influenza A virus (A/H1N1), betacoronavirus-1 (HCoV-OC43) belonging to the same β-coronaviruses as the current pandemic SARS-CoV-2, human herpesvirus type 1 (HHV-1), and human adenovirus type 5 (HAdV-5). Antiviral assays (AVAs) were used to evaluate the antiviral activity of the plant extracts in a cell-present environment with extracts tested before, simultaneously, or after viral infection. The virus replication was assessed using the CPE scale or luminescent assay. The EAM-ESN blend strongly inhibited A/H1N1 replication as well as HCoV-OC43, while having a limited effect against HHV-1 and HAdV-5. This activity likely depends mostly on the presence of the extract of *S. nigra*. However, the EAM-ESN blend possesses more effective inhibitory activity toward virus replication than its constituent extracts. A post-infection mechanism of action of the EAM-ESN make this blend the most relevant for potential drugs and supportive treatments; thus, the EAM-ESN blend might be considered as a natural remedy in mild, seasonal respiratory viral infections.

## 1. Introduction

Influenza virus has been known as a pandemic pathogen since the 16th century [1], but it has probably been around much longer. It circulates in all countries causing a mild to acute disease of the upper and lower respiratory tract, in some cases leading to pneumonia. Many influenza strains are zoonotic viruses originally affecting a wide group of birds and mammals and occasionally transmitting through the species barrier to humans [2]. There are four types of influenza viruses [3], with the most important A and B types causing seasonal illnesses that can turn into pandemics. One of the most serious pandemic, the “Spanish flu”, caused by the influenza A subtype (H1N1), killed 20–40 million worldwide in the years 1918–1920 [4]. Even now, influenza is responsible for approximately 500,000 deaths annually worldwide [3]. In the vast majority of cases, recovery comes within a week without hospitalization or medical drug intervention. The most vulnerable groups to the risk of severe course of disease and complications are the elderly, pregnant women, children, and immunocompromised individuals. There are several drugs that have been accepted over the years to treat influenza infections, such as amantadine, ribavirin, rimantadine, zanamivir, oseltamivir, laninamivir octanoate, peramivir, favipiravir [5], and baloxavir [6], but only oseltamivir phosphate, zanamivir, peramivir, and baloxavir marboxil were approved by the U.S. Food and Drug Administration (FDA) to treat the flu in the 2020/2021 season [7], while the European Medicine Agency (EMA) authorized the use of oseltamivir, peramivir, amantadine, and rimantadine [8]. However, drug-resistant strains of the influenza virus have already occurred, such as the H274Y neuraminidase mutation carried by some strains of the influenza A type resistant to oseltamivir, or the S246R mutation resistant to zanamivir, peramivir, and laninamivir [9]. Fortunately, the global occurrence of drug-resistant influenza strains remains at a low level [9]; nonetheless, influenza virus resistance should be constantly monitored.

Seasonal influenza infections have been recently overshadowed by the emergence of a new respiratory virus belonging to the coronaviruses, which has caused massive infections worldwide. Coronaviruses are a group of RNA viruses belonging to *Nidovirales* and divided into four genera: alpha-, beta-, gamma-, and deltacoronaviruses [10]. Currently, there are seven coronaviruses [11] infecting humans, two alphacoronaviruses species (HCoV-229E and HCoV-NL63), and five members of betacoronaviruses (HCoV-OC43, HCoV-HKU1, MERS-CoV, SARS-CoV-1, and the current pandemic SARS-CoV-2). All of the species that infect people cause respiratory disease with different severity. Among them is SARS-CoV-2 that causes the most dangerous course of the disease, named COVID-19. Only remdesivir has been approved by the FDA and EMA to treat serve COVID-19 in adults and children over 12 years weighing at least 40 kg who required hospitalization [12,13,14]. Anti-COVID-19 drug research is still ongoing.

Although available against several viruses, the development of new antiviral vaccines is not always possible or effective; thus, searching for new antiviral medicines is still needed. A rich source of bioactive compounds are plants that have been traditionally used in the treatment of many diseases for thousands of years. Currently, over 10% of essential drugs available globally have a plant source. The plant extracts appear to be relatively safe, with low side effects that have been observed in contrast to synthetic drugs [15]. Moreover, searching for new medicines is important as drug-resistant viruses as well as frequently mutating viruses such as influenza or coronaviruses might emerge [16], which could possibly become a serious global affair in the near future.

In this paper, the antiviral activity of an original blend of double-standardized extracts of *Aronia melanocarpa* (Michx.) Elliot and *Sambucus nigra* L. (EAM-ESN) against two respiratory viruses, influenza A virus (A/H1N1) and human betacoronavirus-1 (HCoV-OC43), are presented. In addition, the effect of the EAM-ESN blend is assessed against other human respiratory viruses with different structures and belonging to various taxonomic groups: human herpesvirus type 1 (HHV-1) and human adenovirus type 5 (HAdV-5). The effectiveness of the EAM-ESN compared to separated extracts—*A. melanocarpa* (EAM) and *S. nigra* (ESN)—in the inhibition of A/H1N1 and HCoV-OC43 replication was also investigated.

## 2. Results

### 2.1. The Phytochemical Composition of the Extracts

**Figure 1:**
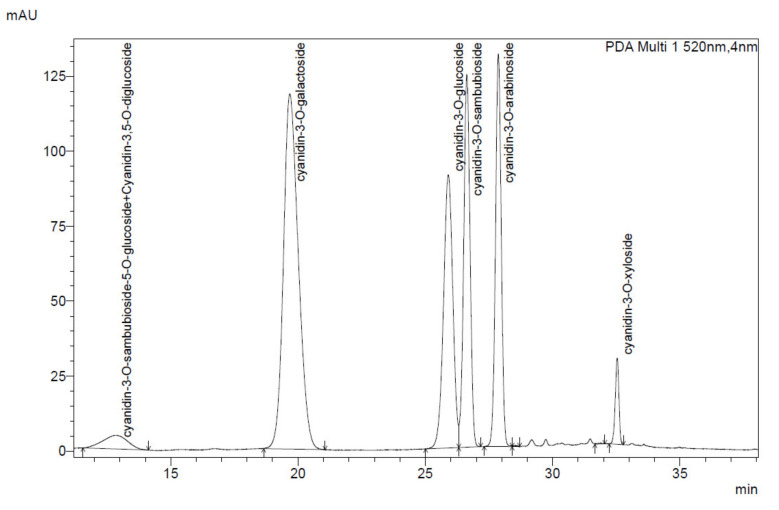
The chromatogram of the EAM-ESN blend with identified anthocyanin compounds (total chromatogram available in Supplementary Materials).

The content of anthocyanins was determined by the HPLC-DAD method. Based on the analysis, we found 28.48% (*m/m*) content of anthocyanins in the EAM-ESN blend, 33.50% (*m/m*) content in the EAM, and 26.15% (*m/m*) in the ESN. The chromatographic analysis for the blend showed the presence of compounds such as cyanidin 3-O-sambubioside-5-O-glucoside, cyanidin-3,5-O-diglucoside, cyanidin 3-O-galactoside, cyanidin 3-O-glucoside, cyanidin 3-sambubioside, cyanidin 3-O-arabinoside, and cyanidin 3-xyloside (Supplementary Material). The chromatogram is shown in Figure 1. The anthocyanin compounds found in the EAM extracts are cyanidin-3-galactoside, cyanidin-3-glucoside, cyanidin-3-arabinoside, and cyanidin-3-xyloside, while in the ESN, we identified cyanidin-3-O-sambubioside-5-O-glucoside, cyanidin-3,5-O-diglucoside, cyanidin-3-O-glucoside, and cyanidin-3-O-sambubioside (Table 1). The total phenolic content was quantified based on the Folin–Ciocalteu assay with catechin as a calibrating curve. The phenolic content was 49.77% (*m/m*) in the EAM-ESN blend, 62.42% (*m/m*) in the EAM, and 43.93% (*m/m*) in the ESN.

### 2.2. Determination of Non-Cytotoxic Concentrations of the Potential Antiviral EAM-ESN Blend

**Figure 2:**
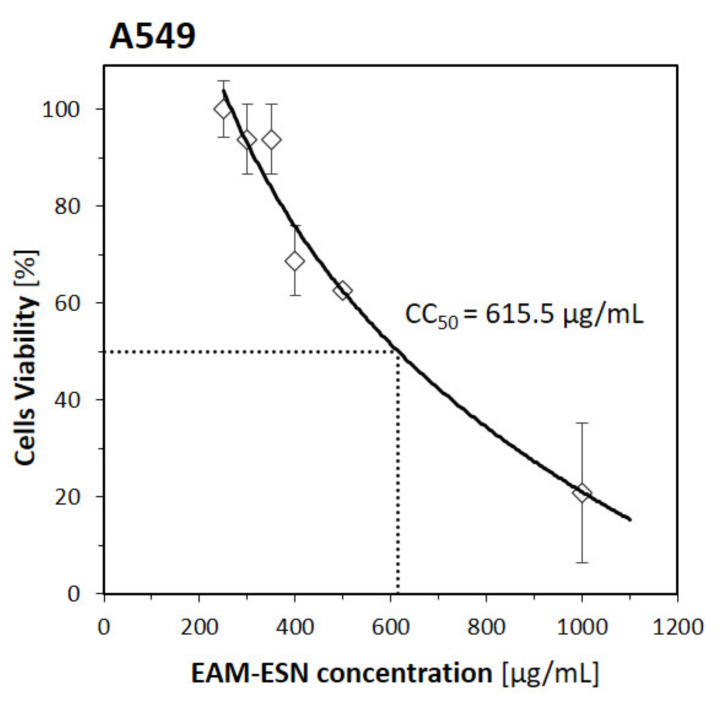
Cytotoxicity of the EAM-ESN blend on A549 cell line with estimated CC_50._ The CC_50_ is calculated based on best-fitting exponential curve. Error bars are CI95% of the means.

In the first step, the non-cytotoxic concentrations (NCCs) of the blend of double-standardized extracts of *A. melanocarpa* and *S. nigra* (EAM-ESN) for antiviral assays were investigated. A549 cells were treated with several concentrations of the EAM-ESN blend (in a range of 100–1000 µg/mL) for 72 h. Next, the cytotoxic effects (CTEs) were evaluated under the inverted microscope. The fresh blend EAM-ESN solution was prepared before each experiment in 50% (*v/v*) DMSO/H_2_O solution. The final DMSO concentration was 1% (*v/v*) with no observable cytotoxic effects. The negative control was untreated cells cultured only with maintenance medium. The experiment was performed three times in at least three independent repetitions. For the A549 cell line treated with the EAM-ESN blend, the NCCs (CTE = 0) were observed for concentrations ≤250 μg/mL. Estimated cytotoxic concentration (CC_50_), a concentration that causes death to 50% of host cells, was CC_50_ = 615.5 μg/mL. The results are shown in Figure 2.

The cytotoxicity assay with estimating CC_50_ for the EAM-ESN blend was also investigated on MDCK (cell line dedicated for A/H1N1) and HCT-8 (cell line dedicated for HCoV-OC43). Minor toxic effects were observed on MDCK with CC_50_ = 1975.3 μg/mL, whereas the EAM-ESN blend resulted in higher cytotoxicity on HCT-8 with CC_50_ = 416.5 µg/mL. Following, NCCs between 100 and 250 μg/mL of the EAM-ESN blend were used for antiviral assays (AVAs) against H1N1, HHV-1, and HAdV-5. Due to higher CTE for HTC-8, tested extracts at the concentration range of 15.6–125 μg/mL were used for AVA against HCoV-OC43.

### 2.3. Antiviral Activity of the EAM-ESN Blend

**Figure 3:**
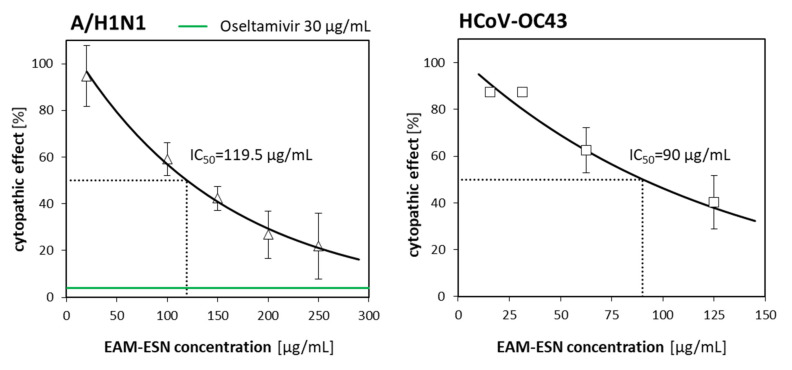
Antiviral effectiveness of the EAM-ESN blend against A/H1N1 and HCoV-OC43. Error bars are CI95% of the means. The IC_50_ is calculated based on the best-fitting exponential curve. The green line is a threshold of positive control (30 µg/mL oseltamivir) shown as a percentage of the cytopathic effect.

Antiviral activity of the EAM-ESN blend against the two most important human respiratory viruses, influenza virus A/H1N1 and coronavirus HCoV-OC43, as well as against two additional viral pathogens, herpesvirus HHV-1 and adenovirus HAdV-5, was examined in vitro in three experimental options (1–3) (see description in *Materials and Methods*). The inhibitory effect was observed in Option 3, when cells were treated with the EAM-ESN blend after infection with H1N1, HHV-1, HAdV-5, or HCoV-OC43. The inhibitory concentration (IC_50_), the concentration inhibiting 50% of virus replication, together with the selectivity index (SI) calculated as a ratio of CC_50_/IC_50_ are presented in Table 2. IC_50_ of the EAM-ESN blend against A/H1N1 was 119.5 μg/mL, and for HCoV-OC43, it was 90.3 μg/mL. The antiviral activity of the EAM-ESN against HHV-1 and HAdV-5 was also indicated: IC_50_ = 292.7 μg/mL and IC_50_ = 498.7 μg/mL, respectively. Results of the AVAs showed that the EAM-ESN blend in a non-toxic concentration of 250 μg/mL inhibited replication of A/H1N1 even by 80%; it also inhibited replication of HHV-1 by 30% and, to smaller extent, HAdV-5 by 10%. At a concentration of 125 μg/mL, the EAM-ESN blend inhibited HCoV-OC43 replication by almost 50% compared to the untreated control. In conclusion, the EAM-ESN blend showed the highest antiviral activity against human influenza virus A/H1N1 and human betacoronavirus HCoV-OC43. The results are presented in Figure 3.

### 2.4. Antiviral Activity of the Individual Plant Extracts EAM and ESN

In the next step, to establish if the EAM-ESN blend presented higher antiviral effects than its constituent extracts, *A. melanocarpa* (EAM) or *S. nigra* (ESN), and which extract was responsible for antiviral activity of the EAM-ESN blend, AVAs were performed. Given the highest antiviral activity of the EAM-ESN blend against A/H1N1 and HCoV-OC43, AVAs with the EAM and ESN were carried out also only with these two viruses. Cytotoxicity of separated extracts *A. melanocarpa* (EAM) and *S. nigra *(ESN) were investigated to set up non-toxic concentrations for experiments against A/H1N1 and HCoV-OC43. CC50 was calculated for each extract on MDCK and HCT-8.

On MDCK cells, the ESN showed no (CTE = 0) or very low (CTE = 1) cytotoxicity for almost all tested concentrations in a range of 50–1000 µg/mL. CC_50_ = 1629.7 µg/mL was estimated (Table 3). The EAM presented no (CTE = 0) or very low (CTE = 1) cytotoxicity for concentrations of ≤250 µg/mL. Concentrations of ≥500 µg/mL expressed high toxicity (CTE = 3–4). CC_50_ = 838.9 µg/mL was estimated (Table 3). Following, the highest NCCs of the ESN 250 µg/mL and EAM 250 µg/mL were used for AVA experiments against A/H1N1.

HCT-8 cells were much more sensitive to the tested extracts. The ESN showed no (CTE = 0) or very low (CTE = 1) cytotoxicity for concentrations ≤250 µg/mL with estimated CC_50_ = 475.2 µg/mL (Table 3). However, the EAM presented high toxicity. The lack of (CTE = 0) or very low (CTE = 1) cytotoxicity was observed only for concentrations of ≤31.25 µg/mL. CC_50_ = 102.2 µg/mL was estimated (Table 3). Following, the highest NCCs of the ESN 250 µg/mL and the EAM 31.25 µg/mL were used for AVA experiments against HCoV-OC43.

**Figure 4:**
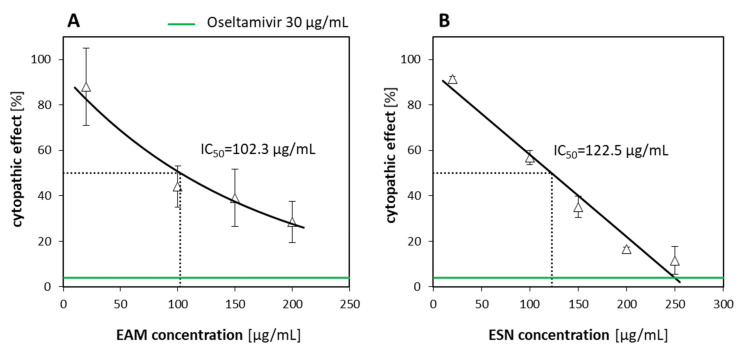
Antiviral effectiveness of EAM (**A**) and ESN (**B**) against A/H1N1. Error bars are CI95% of the means. The IC_50_ is calculated based on the best-fitting exponential or linear curve. The green line is a threshold of positive control (30 µg/mL oseltamivir) shown as a percentage of the cytopathic effect.

Results of antiviral activity tests showed that both separated extracts EAM and ESN exhibited inhibitory activity against the influenza virus. The EAM inhibited A/H1N1 replication by 72% at NNCs (IC_50_ = 102.3 µg/mL), while the ESN inhibited A/H1N1 replication by over 80% (IC_50_ = 122.5 µg/mL). The results are presented in Figure 4. Interestingly, neither EAM nor ESN expressed any significant antiviral activity against HCoV-OC43. The observed decline in replication was below 15% for the ESN and below 8% for the EAM (Table 3).

## 3. Discussion

Countless diseases of bacterial, fungal, and viral origin showed how crucial it is to search and develop new medicines against the pathogens that cause them. In the case of viral infections, various antiviral drugs are available. However, still, the most important issues are the occurrence of drug-resistant viruses, viruses with high variability, as well as the identification of new viral strains with no accessible antiviral drugs. The answer could be combinatory therapies [17] using purified natural products or their blends. Traditional medicine allows us to take advantage of a generous source of plant compounds. Over the past decade, natural products have helped to combat many viral agents, including respiratory viruses. Plant extracts or combining extracts gives the possibility to increase the spectrum of antiviral activity to more virus species. There are numerous findings that confirm the effectiveness of plant-derived substances against many diseases, including infectious ones [18]. Different extracts of black elderberry (*S. nigra*) [19,20] and black chokeberry (*A. melanocarpa*) [21] are known for their health-promoting antimicrobial properties that have been used for a long time in folk medicine and in the food industry. These plants owe their properties mainly to the high content of various polyphenols [22,23,24]. Here, we demonstrate the antiviral activity of an originally developed blend of double-standardized extracts of *A. melanocarpa* and *S. nigra* (EAM-ESN) and its individual components against the human respiratory viruses influenza virus A/H1N1 and betacoronavirus HCoV-OC43, belonging to the β-coronaviruses as the current pandemic SARS-CoV-2. The use of a mixture of these two extracts can increase the effectiveness of the preparation due to the complementary action of the various anthocyanins that each extract contains [22,23]. This synergistic effect can have significant benefits by reducing the amount of extract ingested while maintaining its health-promoting properties or even extending its scope of antimicrobial activity. Such a combinatory effect was seen in different studies. An additive antiviral effect of black currant anthocyanins against influenza A and B virus was found by Knox et al., 2001 [25], while synergistic antiviral effects against HHV-1 have also been reported for flavones and flavonols [26]. Musarra-Pizzo et al. [27] have shown that polyphenols extracted from pistachios are effective against HHV-1, assuming this effect as a result of a balance of the individual polyphenolic components.

Coronavirus infections and influenza share similar “influenza-like” clinical manifestations usually of mild or moderate course. However, simultaneous infections might result in a big challenge in controlling both diseases, as it is currently observed for the COVID-19 pandemic and seasonal influenza [24]. Betacoronaviruses and influenza viruses are droplet-borne pathogens that invade the upper or lower respiratory tract by binding to specific cell surface receptors [28]. In severe cases, these viruses might cause pneumonia and other serious complications, especially in the presence of bacterial infections. Importantly, co-infection may occur easily [24]. Natural compounds would provide considerable support to encourage the course of the disease and healing process. Good candidates for antiviral agents should have the ability to block virus entry on cell surfaces and/or block intracellular replication in host cells. Our results demonstrated that the EAM-ESN blend inhibited viral replication when it was administrated after infection with influenza A/H1N1 or coronavirus HCoV-OC43. We suggested that the EAM-ESN might inhibit the multiplication of the A/H1N1 by influencing its replication cycle or release of the virus from infected cells. This is an RNA virus containing an envelope with two glycoproteins: hemagglutinins (HA) and neuraminidase (NA). The antiviral activity towards neuraminidase inhibition seems to be the most likely. Some anthocyanins might interact with neuraminidase and prevent the release of virions [29]. The interaction of cyanidin-3-sambubiocid with neuraminidase was simulated [30], showing high sensitivity and potential antiviral activity for wild and mutant (H274Y) types of this peptide. Representatives of anthocyanidins, which are the sugar-free anthocyanins, such as luteolinidin, apigeninidin, or luteolin, were also found to inhibit neuraminidase [31]. The EAM-ESN blend is double-standardized to at least 25% of anthocyanins and 40% of polyphenols, which may be responsible for the main activity of the compound. It is believed that the high amount of anthocyanins and polyphenols is a key advantage of this blend. Antiviral properties of different polyphenols were widely presented. Polyphenolic extracts can inhibit viruses of various origins. The synergistic interaction of polyphenolics was especially underlined, which can improve the inhibiting effect of these compounds [32]. It was presented by Mohammadi Pour et al. [33] that anthocyanins are among the critical supplements that improve human health. Their potential antiviral effects express through binding to host cells, inhibiting the viral life cycle, or stimulating host immunity. The betacoronavirus-1 was less sensitive to the EAM-ESN antiviral activity. Possibly, the EAM-ESN target was different for HCoV-OC43 but was located between the replication cycle and cell release process. Recently, Kim et al. [34] highlighted that potential candidates for inhibition in the SARS-CoV-2 life cycle are S-glycoprotein receptor, cellular protease, virus protease, RNA polymerase, inflammasome, RNA helicase, and SA-O-Ac-esterase. Naturally occurring compounds, such as silvestrol, baicalin, saikosaponin, linolenic acid, quercetin, kaempferol, wogonoside, curcumin, and many other aqueous extracts are promising antiviral agents [34]. The authors pointed out that SARS-CoV-2 targeting agents can be effective also against other coronaviruses due to the similarity in life cycles. The observed antiviral activity of an originally developed composition EAM-ESN should help to limit the spreading of both viruses and the development of the infection. This mechanism of action is the most relevant for potential drugs and supportive treatments; thus, the EAM-ESN blend might be considered as a natural remedy in mild, seasonal respiratory viral infections. Moreover, many in vitro and in vivo studies have shown that polyphenols support the body’s immune functions, acting as anti-inflammatory and immunomodulatory agents [32]. Thus, blends with high polyphenol content may present desirable effects in diminishing viral infections. Bioavailability has a significant impact on the effectiveness of natural origin preparations. To become bioactive, most flavonoids must undergo several chemical transformations through digesting or microbiota metabolism processes such as sugar or hydroxyl moieties tailoring [35]. Then, the properly prepared compounds can be absorbed in the intestines and circulated via blood to distant organs [35]. Recovery of anthocyanins in ileal fluid measured 4 h after consumption of 300 g raspberries showed 75 µmol anthocyanins (i.e., cyanidin-3-O-glucoside or cyanidin-3-O-rutinoside) in total [36]. However, no raspberry-derived anthocyanins were detected in the plasma of the healthy subjects [36]. In different studies [37,38], intake of 720 mg of anthocyanins from elderberry extract in total resulted in 97 nmol anthocyanin concentrations measured in the plasma 1 h after ingestion. The bioavailability of the various polyphenols varies considerably, but it seems most anthocyanins might be poorly absorbed [39]. Certainly, in vivo studies of the blend and its constituent extracts are needed to determine the bioavailability, metabolism, and effectiveness of the antiviral activity. Additionally, the future challenges are studies of the EAM-ESN main mechanisms of action. However, we can already conclude that the tested extracts are more active against enveloped viruses.

The antiviral activities of the blend towards herpes virus (HHV-1) and human adenovirus (HAdV-5) were weak with extrapolated inhibitory activity on 292.7 µg/mL and 498.7 µg/mL, respectively. Taking into consideration the poor selectivity index (2.1 for HHV-1, and 1.2 for HAdV-5) and based on our experience, we assumed that the antiviral activity for constituent extracts would be similar. Therefore, we focused on the most potent viruses, influenza (A/H1N1) and coronavirus-1 (HCoV-OC43), that are also more important from an epidemiological perspective. It seems our assumptions were confirmed by observation for A/H1N1 and HCoV-OC43, where selectivity indexes were weaker for EAM and ESN, with the most distinct differences for HCoV-OC43.

Considering the antiviral activity of the EAM-ESN blend against human influenza A virus and human betacoronavirus-1, our next question was which of the separated constituents was responsible for this effect. The effectiveness of the EAM-ESN blend with individual extracts of *A. melanocarpa* (EAM) and *S. nigra* (ESN) was evaluated. Based on the obtained selectivity indexes, we concluded that the original EAM-ESN blend showed better antiviral activity against A/H1N1 and HCoV-OC43 than its constituent extracts (Table 4). In addition, we showed that the antiviral activity of the EAM-ESN blend against A/H1N1 (SI = 16.5) was mainly due to the content of the ESN (SI = 13.3). In AVA experiments, ESN showed strong properties to inhibit A/H1N1 replication. Additionally, the ESN was characterized by very low toxicity towards human cells. There are many studies that have investigated the antiviral properties of pure elderberry extracts. Roschek et al. [40] found that flavonoids from elderberry bind directly to influenza (A/H1N1) virions, preventing infection of the host cell in vitro. The authors identified two possible flavonoid compounds (5,7-dihydroxy-4-oxo-2-(3,4,5-trihydroxyphenyl)chroman-3-yl-3,4,5-trihydroxycyclohexanecarboxylate and 5,7,30,40-tetra-O-methylquercetin) that could be responsible for the antiviral activity of the elderberry extract [40]. It is worth mentioning that they found the antiviral activity through direct incubation of the extract with A/H1N1, while in our experiments, we did not observe direct antiviral activity during incubation of all tested extracts with viruses A/H1N1, HCoV-OC43, HHV-1, and HAdV-5 in a cell-free state, either (data not shown). Different preparation procedures and final contents of the extracts may also influence their antiviral properties and mechanism of action. The extract from *S. nigra* used in our experiments was extracted within water and ethanol solvents, while the extract used by Roschek Jr. et al. was obtained by supercritical CO_2_ extraction. For instance, Zhou et al. [41] compared different extraction methods of *Cinnamomi ramulus* for its antiviral activity against human herpesvirus 1 (HHV-1) and respiratory syncytial virus (RSV) in vitro. Their results demonstrated that there are differences between ethanol extraction, hydrodistillation, supercritical fluid extraction, and reflux extraction in cytotoxicity and inhibitory activity [41]. In other studies, Krawitz et al. [42] tested standardized elderberry liquid extract against influenza A and B with 30% and 25% foci size reductions, respectively. Kinoshita et al. [43] showed weak anti-influenza activity of elderberry juice in vitro when the extract was administrated to cells during and after infection with influenza A virus, A/NWS/33. However, they observed prevention of the viral infection after oral administration of the high-molecular-weight fraction of concentrated elderberry juice to infected mice [43]. In contrast, our results indicated that the EAM showed lower antiviral activity against A/H1N1 with higher toxicity; hence, we concluded that the EAM-ESN blend probably owes its toxicity to the EAM component. There are limited data showing antiviral activity of the extract of *A. melanocarpa*. In our study, the individual *A. melanocarpa* extract (EAM) showed the weakest potency as an inhibitory compound against A/H1N1 and HCoV-OC43. Interestingly, in a different study, the ethanol extract from crude *A. melanocarpa* plant exhibited strong broad-spectrum activity against various influenza A and B viruses in a range of 0.0625–1 mg, including an oseltamivir-resistant one [44]. Our results showed that the extract of *A. melanocarpa* undeniably performed better against A/H1N1 than against betacoronavirus-1. However, comparing selectivity indexes, the antiviral activity was approximately two times weaker for *A. melanocarpa* than for *S. nigra* and the blend of these two.

The highest non-toxic concentration of the EAM-ESN (250 µg/mL) inhibited replication of the influenza virus by 80%. To obtain 80% of A/H1N1 inhibition by the individual ESN, concentrations above 200 µg/mL must be used. The content of elderberry (ESN) and black chokeberry (EAM) extracts in the EAM-ESN blend was in a 2:1 ratio. This means that lower concentrations of the ESN with an addition of the EAM allowed for maintaining high antiviral activity of the EAM-ESN blend, as was observed for separated ESN, but in much higher concentrations. It is an important feature of the EAM-ESN blend considering the fact that it still maintained low toxicity. This relation was even more distinct for betacoronavirus-1. The *A. melanocarpa* and *S. nigra* extracts showed no antiviral activity (SI = 0.8 and SI = 0.9, respectively), while the EAM-ESN blend decreased HCoV-OC43 virus replication by 50% (SI = 4.6) compared to the untreated control (Table 4). The reduction in the toxicity while retaining the antiviral activity is the strongest advantage of the originally created composition EAM-ESN.

## 4. Materials and Methods

### 4.1. Cell Lines

Human epithelial carcinoma cells (A549; ATCC^®^ CCL-185™) were cultured with Dulbecco’s Modified Eagle Medium (DMEM; Hirszfeld Institute of Immunology and Experimental Therapy Polish Academy of Sciences (HIIET PAS), Wrocław, Poland) with 5% (*v/v*) fetal bovine serum (FBS; Sigma-Aldrich, Steinheim, Germany).

Madin–Darby canine kidney cells (MDCK; ATCC^®^ CCL-34™) were cultured with Eagle’s Minimum Essential Medium (EMEM; HIIET PAS, Wrocław, Poland) with 5% (*v/v*) FBS.

Human ileocecal adenocarcinoma cells (HCT-8 [HRT-18]; ATCC^®^ CCL-244™) were cultured with Roswell Park Memorial Institute 1640 Medium (RPMI-1640 Medium; HIIET PAS, Wrocław, Poland) with 10% (*v/v*) FBS.

Growth media for A549, MDCK, and HCT-8 were also supplemented with antibiotics (100 U/mL penicillin and 100 μg/mL streptomycin) and 2 mM L-alanyl-L-glutamine (all provided by Corning, Manassas, VA, USA). Cells were cultured at 37 °C in a humidified 5% CO_2_ atmosphere in a New Brunswick Galaxy 170 S incubator (Eppendorf, Hamburg, Germany). Cells were harvested twice a week with Trypsin/EDTA solution (HIIET PAS, Wrocław, Poland) divided and placed into a new flask with a fresh growth medium.

### 4.2. Viruses

Human influenza A virus (A/H1N1, A/PR/8/34 strain; *Orthomyxoviridae*, ATCC VR-1469™) was cultured on MDCK cells in a maintenance medium (EMEM supplemented with 1 mM 4-(2-hydroxyethyl)-1-piperazineethanesulfonic acid (HEPES), 1 µg/mL TPCK-treated trypsin (Thermo Scientific, Rockford, IL, USA), 0.1275% (*v*/*v*) BSA fraction V (Gibco, Grand Island, NY, USA), 2 mM L-glutamine, and antibiotics of 100 U/mL penicillin and 100 μg/mL streptomycin).

Human betacoronavirus OC43 (HCoV-OC43; *Coronaviridae*, ATCC VR-1558™) was cultured on HCT-8 cells in maintenance medium (RPMI 1640 Medium supplemented with antibiotics (100 U/mL penicillin and 100 μg/mL streptomycin and 2 mM L-glutamine).

Human alphaherpesvirus 1 (HHV-1, MacIntyre strain; *Herpesviridae*, ATTC VR-539™) and Human adenovirus 5 (HAdV-5, Adenoid-75 strain; *Adenoviridae*, ATCC VR-5™), HHV-1 and HAdV-5, were cultured on A549 cells in a maintenance medium (DMEM supplemented with antibiotics (100 U/mL penicillin and 100 μg/mL streptomycin, 2 mM L-glutamine, and 2% FBS).

Virus titers were expressed as a tissue culture infectious dose per 0.1 mL (TCID_50_/0.1 mL) with a 50% endpoint where half of the inoculated host cells produced a cytopathic effect (CPE) and were calculated using the Spearman–Kärber method [45]. The CPE of HCoV-OC43 was visualized using immunoperoxidase dying. Any media used for virus culturing and experiments are collectively referred to as *maintenance medium* throughout the paper.

### 4.3. Blend and Extracts

An original blend of double-standardized extracts of black chokeberry (*A. melanocarpa*) and elderberry (*S. nigra*) (EAM-ESN, proprietary composition—*Fenactive*), as well as separated extracts of *A. melanocarpa* (EAM) and *S. nigra* (ESN) were provided by Greenvit Botanical Extracts Manufacturer, Zambrów, Poland. The blend was prepared by the manufacturer by mixing 2 parts elderberry extract and 1 part black chokeberry extract (2:1 ratio). The EAM-ESN was standardized for polyphenols ≥40% and anthocyanins ≥25%.

### 4.4. Extraction Procedure

Frozen elderberry and black chokeberry fruits were water-extracted at 45 °C for 1 h with percolation and then filtered. The drug/extract ratio (DRE) was 70:1 for elderberry extract and 60:1 for black chokeberry. The total content of anthocyanins and phenolic acids was determined by HPLC-DAD and the Folin–Ciocalteu method. To obtain approx. 400 g of anthocyanins, more than 700 L of elderberry extract with 566.6 mg/L anthocyanin concentration or more than 600 L of black chokeberry extract with 657.4 mg/L anthocyanin concentration were loaded on a chromatography column with adsorption resin. The column was washed with water, and the adsorbed compounds were eluted using 75% ethanol. The main fraction from the column was concentrated by applying a vacuum evaporator to 25% of the dry weight. Afterwards, both extracts were dried on a spray dryer.

### 4.5. Identification and Quantification of Anthocyanin and Phenolic Acids by HPLC-DAD Method

The identification and quantification of anthocyanins and phenolic acids of black chokeberry and elderberry extracts were carried out using a Shimadzu High-Performance LC system equipped with a photodiode array detector (Shimadzu Corp., Kyoto, Japan). The solid extracts were dissolved in 40% methanol to obtain a concentration of approx. 1 mg/mL. The separations were carried out by injecting a 10 µL sample onto a Kinetex Evo C18, 4,6/250 column (Phenomenex, Torrance, CA, USA) at 25 °C. The mobile phase consisted of solvent A (4.5% formic acid, *v*/*v*) and solvent B (100% acetonitrile) (Table 5).

The anthocyanin content was calculated as cyanidin-3-O-glucoside chloride (C3G) equivalent. Anthocyanins acids were identified based on the characteristic UV-Vis spectrum with absorbance maxima at about 520 nm and 280 nm. The identification of anthocyanin compounds in elderberry and black chokeberry extracts was conducted based on characteristic anthocyanin profiles known in the literature [46,47,48,49]. Cyanidin-3-O-glucoside attendance was confirmed with the commercially available standard (Extrasynthese, Genay, France).

### 4.6. Folin–Ciocalteu Assay

The total phenolic content was determined utilizing the Folin–Ciocalteu method with catechin as a standard (Extrasynthese, Genay, France) using a 5-point calibration curve. Then 0.1 g of extract was dissolved in 50 mL of 80% methanol. Subsequently, 1 mL of extract was added to 4 mL of water and mixed with 0.5 mL of the Folin–Ciocalteu reagent (Chempur, Piekary Śląskie, Poland). After 1 min, 2 mL of 20% (*w*/*v*) sodium carbonate aqueous solution was added to a 10 mL volumetric flask which was then filled up with water to a mark. The samples were incubated at room temperature in the dark for 30 min. Then, the absorbance of the solution was measured spectrophotometrically at 760 nm using a UV-1900 spectrophotometer (Shimadzu Corp., Kyoto, Japan). All analyses were carried out in triplicates.

### 4.7. Extract Solutions

Before each experiment, the tested extracts were dissolved in 50% (*v*/*v*) dimethyl sulfoxide (DMSO; Sigma, St. Louis, MO, USA) and water at a concentration of 100 mg/mL as a starting stock and mixed thoroughly until complete dissolution. Next, serial dilutions were performed with a maintenance medium to obtain the final concentrations of 7.8–2500 μg/mL used in experiments.

### 4.8. CTE Cell Viability Assay

A549, MDCK, or HCT-8 were seeded into 96-well microplates at densities of 2.0 × 10^4^ cells/well, 3.0 × 10^4^ cells/well, and 3.0 × 10^4^ cells/well, respectively, and incubated overnight at 37 °C with 5% CO_2_ to reach at least 90% confluence. Growth media were discarded and cells were treated with several concentrations in a range of 50–2000 µg/mL of tested extracts for 72–96 h at 37 °C, 5% CO_2_. The viability was measured by evaluating morphological changes observed by an inverted microscope assessed by a four-point cytotoxic effects (CTEs) scale, where 0—lack of visible CTEs in cells; 1—CTEs in up to 25% of cells; 2—CTEs in up to 50% of cells; 3—CTEs in up to 75% of cells; and 4—CTEs in up to 100% of cells. The negative control was untreated cells cultured only with maintenance medium.

### 4.9. MTT Assay

For the MTT assay, 96-well microplates with 3.0 × 10^4^ cells/well of MDCK and HCT-8 cell lines were prepared. After overnight incubation at 37 °C with 5% CO_2_, growth media were discarded. Next, cells were treated with test extracts at concentrations in a range of 15.6–2500 µg/mL for 72–96 h at 37 °C, 5% CO_2_. The maintenance medium above cells was discarded, cells were rinsed once with PBS, and fresh medium was added. Next, 20 μL of 5 mg/mL MTT (Sigma, St. Louis, MO, USA) solution was added to each well and the plate was incubated at 37 °C, 5% CO_2_ for 3 h, then 100 μL of 10% (*m*/*v*) SDS (Sigma, St. Louis, MO, USA) with 1 N HCl (Chempur, Piekary Śląskie, Poland) solution was applied to each well. The absorbance was read at 570 nm wavelength with the microplate reader Multiskan RC (Thermo Labsystem, Vantaa, Finland) and compared to the untreated control.

### 4.10. Antiviral Assays (AVAs)

Antiviral assays (AVAs) were used to evaluate the antiviral activity of the tested plant-derived extracts in a cell-present environment. Three different experiments were designed. First (Option 1), to check biological activity to excite an antiviral state, cells were incubated with EAM-ESN, EAM, or ESN 24 h before infection. Second (Option 2), to assess the inhibition by the extracts of virus ability to attach or enter host cell, cells were incubated with EAM-ESN, EAM, or ESN simultaneously with the virus. Third (Option 3), to determine the influence of the tested extracts on the replication cycle of the virus, cells were incubated with EAM-ESN, EAM, or ESN after viral infection. A549, MDCK, or HCT-8 cells were treated with the tested extracts in several concentrations in a range of 7.8–250 μg/mL and infected with 100 TCID_50_/0.1 mL of virus inoculum. The adsorption time was 30 min for H1N1, 40 min for HHV-1, and HAdV-5 or 90 min for HCoV-OC43 at a virus-specific temperature: 33 °C for H1N1 and HCoV-OC43 or 37 °C for HHV-1 and HAdV-5 and 5% CO_2_ atmosphere. After adsorption, the maintenance medium was discarded and cells were rinsed by PBS twice and fresh maintenance medium (Options 1 and 2) or preparation solution (Option 3) were added to the cells. Positive controls were 30 µg/mL oseltamivir (Sigma-Aldrich, St. Louis, MO, USA) for H1N1, 100 µg/mL cidofovir (Cayman Chemical Company, Ann Arbor, MI, USA) for HHV-1, and 100 µg/mL ribavirin (EDQM, Strasbourg, France) for HAdV-5. Currently, effective anti-CoV agents are not available; thus, the positive control for HCoV-OC43 was not implemented in this study. Negative controls were cells treated only with viruses in maintenance medium. Plates were incubated for 2–4 days, and viral cytopathic effects (CPE) were evaluated.

### 4.11. Microscopic Viral Cytopathic Effects (CPE)

Viral cytopathic effects (CPE) were observed and evaluated under the inverted microscope with a five-point CPE scale: 0—lack of viral replication (lack of CPE), 1—viral CPE in 25% of cells, 2—viral CPE in 50% of cells, 3—viral CPE in 75% of cells, and 4—100% of the cells affected with CPE. Viral titer was expressed with reference to the TCID50.

### 4.12. Luminescent Assay for Viral-Induced CPE

To determine viral CPE and monitor tested extracts for antiviral activity, the luminescent Viral ToxGlo™ kit (Promega, Madison, WI, USA) assay was performed according to the manufacturer’s protocol. The MDCK cell line was seeded on a 96-well microplate at 3.0 × 10^4^ cells/well and incubated 24 h at 37 °C, 5% CO_2_. Next, cells were treated with 100 TCID_50_/0.1 mL of H1N1 inoculum and several concentrations (20–250 μg/mL) of the EAM-ESN blend and separated extracts of the EAM or ESN for 72 h at 33 °C, 5% CO_2_. After reaching 100% of CPE in the negative control, the maintenance medium was discarded and a fresh one was added. The luminescence was measured with Glomax Luminometer (Promega, Sunnyvale, CA, USA) and compared to the untreated cell control.

### 4.13. Immunoperoxidase Assay

To better visualize CPE of HCoV-OC43, an indirect immunoperoxidase assay (IPA) was introduced [50]. The HCT-8 cells were seeded on a 96-well microplate at a density of 3.0 × 10^4^ cells/well. Upon reaching at least 90% confluence, cells were treated with several concentrations 7.8–250 μg/mL of the EAM-ESN blend with HCoV-OC43 inoculum for 4 days at 33 °C and 5% CO_2_. Next, the supernatants were discarded and the monolayers were rinsed once with PBS. To visualize HCoV-OC43 CPE, the cells were overlayed by the primary mouse anti-coronavirus monoclonal antibodies (Merck, Darmstadt, Germany) and secondary goat anti-mouse antibodies conjugated to horseradish peroxidase (Thermo Fisher, Rockford, IL, USA). A virus-specific presence color reaction was obtained by adding a 3,3′-Diaminobenzidine (DAB/H_2_O_2_) solution (Sigma, St. Louis, MO, USA). Stained cells were observed under the inverted microscope to evaluate virus replication in host cells according to the CPEs scale.

### 4.14. Statistical Analysis

The viability of the host cell was measured by two different methods according to its applicability. The CTE cell viability was applied on the A549 cell line and observed based on the CTEs scale. We have shown earlier that this method is widely accepted and resulted in high reproducibility and consistency with different, more sophisticated methods [51]. The percentage of viability was calculated with the formula %viability=−25×avg.CTE+112.5. The viabilities of the MDCK and HCT-8 cell lines based on MTT assay were calculated based on absorbance from nine individual repetitions using the formula of $\%viability=\frac{avg.Abs_{tested}-avg.Abs_{blank}}{avg.Abs_{control}-avg.Abs_{blank}}\times 100\%$. The cytotoxicity concentration and inhibitory concentration were expressed as the 50% cytotoxic concentration (CC_50_ μg/mL) and the 50% inhibitory concentration (IC_50_ μg/mL) and calculated with the general formula for linear regression $f\left(x\right)=ax+b$, exponential regression $f\left(x\right)=ae^{bx}$, or logarithmic regression $f\left(x\right)=a\ln\left(x\right)+b$ based on the best model fit. The CPE of the influenza virus was measured by the luminescence method with three independent repeats with four replicates each. The weighted average of the means from each repetition was calculated with variance as a weight. To calculated the percentage of CPE, the following formula was applied: $\%CPE=\frac{avg.LUM_{tested}-avg.LUM_{control}}{avg.LUM_{H1N1}-avg.LUM_{control}}\times 100\%$. The CPE for betacoronavirus-1 were assessed after immunoperoxidase dying by the CPE method as well for human herpesvirus and human adenovirus, where the percentage of cytopathic effect was calculated according to the formula %CPE=25×avg.CPE−12.5.

## 5. Conclusions

In conclusion, the original blend of double-standardized extracts of *A. melanocarpa* and *S. nigra* (EAM-ESN) strongly inhibited human influenza A virus (A/H1N1) replication as well as human betacoronavirus-1 (HCoV-OC43) at completely non-toxic concentrations. This activity likely depends mostly on the presence of the extract of *S. nigra* (ESN) and less on the extract of *A. melanocarpa* (EAM). Nevertheless, the specific proportion of EAM to ESN enables the blend to keep its high antiviral activity. This activity is even more noticeable against betacoronavirus-1, where individual components of the blend showed no antiviral activity in contrast to the significant activity of the blend. It is worth emphasizing that EAM-ESN will certainly not replace available antiviral drugs; nonetheless, considering the low toxicity of the blend in vitro, we can assume its safety for use. However, further in vivo research is needed to determine the applicability of this blend and to evaluate the effectiveness of its antiviral activity on humans.

## Figures and Tables

**Table 1:** Anthocyanin compounds identified in extracts based on the HPLC-DAD method.

Extracts	Anthocyanin Compounds
Extract of *A. melanocarpa*; EAM	cyanidin-3-galactoside, cyanidin-3-glucoside, cyanidin-3-arabinoside cyanidin-3-xyloside
Extract of *S. nigra*; ESN	cyanidin-3-O-sambubioside-5-O-glucoside, cyanidin-3,5-O-diglucoside, cyanidin-3-O-glucoside cyanidin-3-O-sambubioside
EAM+ESN	cyanidin 3-O-sambubioside-5-O-glucoside cyanidin-3,5-O-diglucoside cyanidin 3-O-galactoside cyanidin 3-O-glucoside cyanidin 3-sambubioside cyanidin 3-O-arabinoside and cyanidin 3-xyloside

**Table 2:** Antiviral activity of the EAM-ESN blend against H1N1, HCoV-OC43, HHV-1, and HAdV-5.

Virus	Genome	Outer Lipid Layer	Cell Line	EAM-ESN Blend (Extract of *A. melanocarpa* + Extract of *S. nigra*)		
				CC_50_	IC_50_	SI
				μg/mL	μg/mL	
A/H1N1	ssRNA(-)	+	MDCK	1975.3	119.5	16.5
HCoV-OC43	ssRNA(+)	+	HCT-8	416.5	90.3	4.6
HHV-1	dsDNA	+	A549	615.5	292.7	2.1
HAdV-5	dsDNA	−	A549	615.5	498.7	1.2

**Table 3:** Antiviral activity of EAM and ESN against H1N1 and HCoV-OC43.

Virus	Cell Line	Extract of *A. melanocarpa*; EAM			Extract of *S. nigra*; ESN		
		CC_50_	IC_50_	SI	CC_50_	IC_50_	SI
		μg/mL	μg/mL		μg/mL	μg/mL	
A/H1N1	MDCK	838.9	102.3	8.2	1629.7	122.5	13.3
HCoV-OC43	HCT-8	102.2	119.9	0.8	475.2	523.1	0.9

**Table 4:** Comparison of the selectivity indexes of the EAM-ESN, EAM, and ESN.

Virus	The EAM-ESN	The EAM	The ESN
	SI	SI	SI
A/H1N1	16.5	8.2	13.3
HCoV-OC43	4.6	0.8	0.9
HHV-1	2.1	-	-
HAdV-5	1.2	-	-

**Table 5:** Mobile phase gradient used for the chromatographic method.

Time (min)	%A	%B	Flow (mL/min)
0–15	94	6	1
18	93	7	0.8
30	82	18	0.8
35	80	20	0.8
38	5	95	0.8
43	94	6	1
48	94	6	1

## Data Availability

Data is contained within the article and Supplementary Material.

## Supplementary Materials

The following supporting information can be downloaded at: https://www.mdpi.com/article/10.3390/ph15050619/s1, Figure S1: The total chromatogram of the EAM-ESN blend, EAM, and ESN.
