# Supplementary material for: Inhibition of Human Respiratory Influenza A Virus and Human Betacoronavirus-1 by the Blend of Double-Standardized Extracts of Aronia melanocarpa (Michx.) Elliot and Sambucus nigra L
Source: Pharmaceuticals (Basel). 2022 May 17;15(5):619. doi: 10.3390/ph15050619 (PMC9143272; doi:10.3390/ph15050619)

==== Shimadzu LabSolutions Multi-Chromatogram ====

The chromatogram of EAM

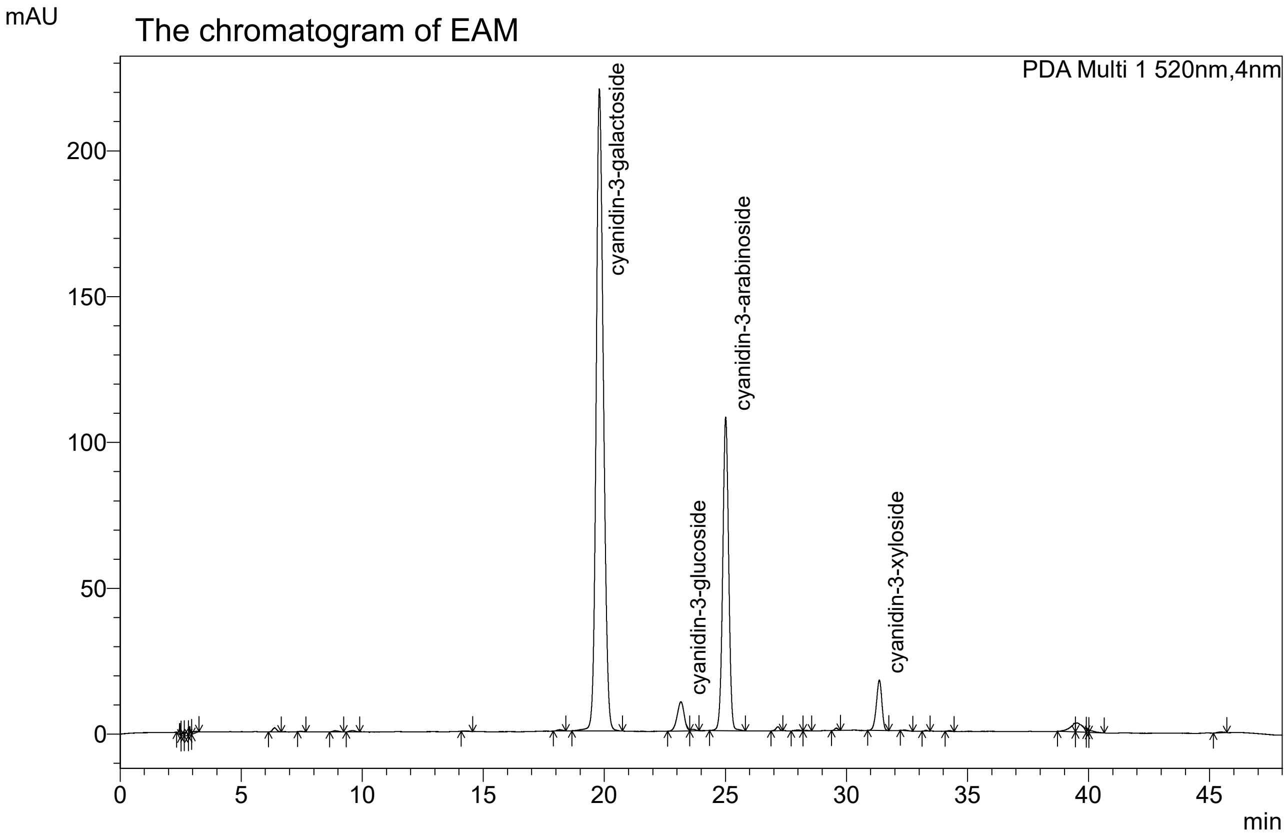

==== Shimadzu LabSolutions Multi-Chromatogram ====

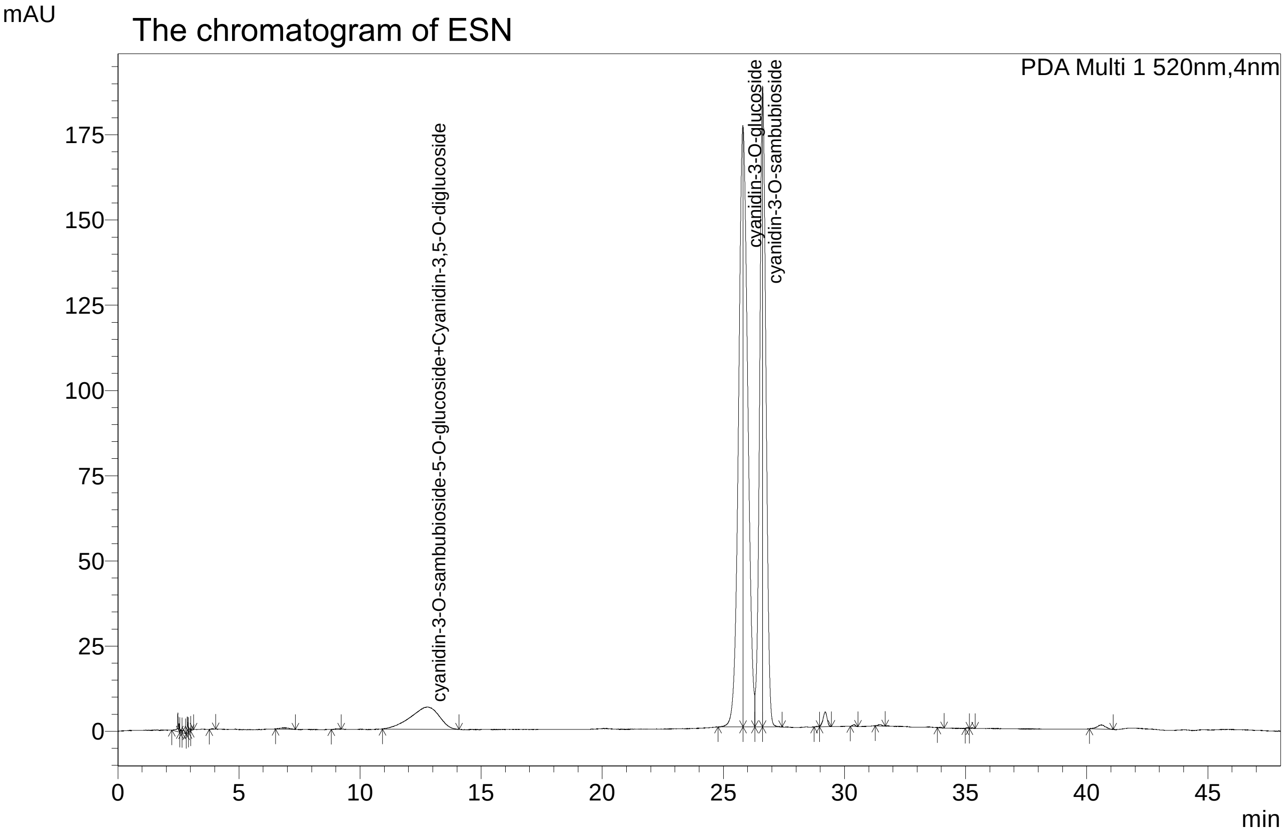

==== Shimadzu LabSolutions Multi-Chromatogram ====

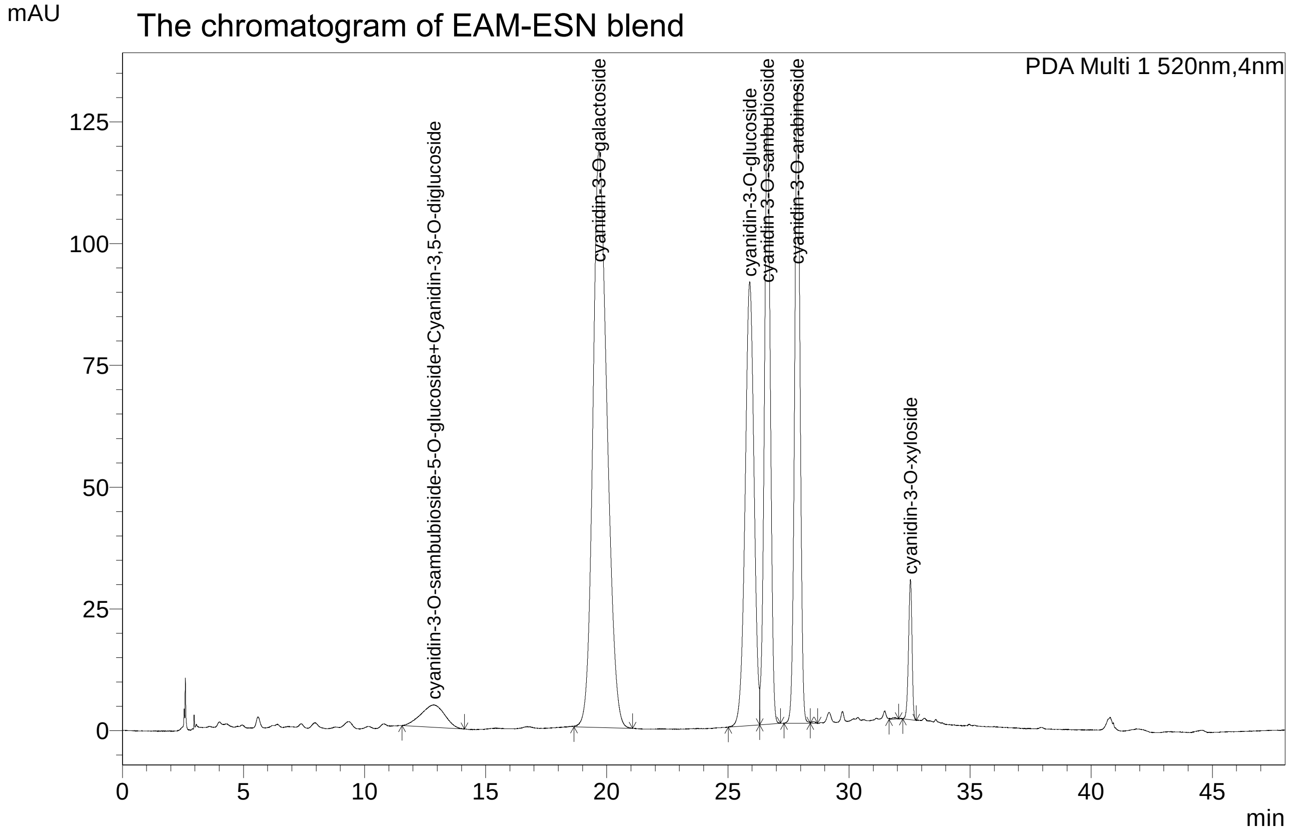

Supplement: Supplementary file 1 [file pharmaceuticals-15-00619-s001.zip › pharmaceuticals-1684715-supplementary.pdf]
